# Shaking in Darkness: Posterior Reversible Encephalopathy Syndrome (PRES)-Induced Acute Bilateral Blindness and Seizures

**DOI:** 10.7759/cureus.97942

**Published:** 2025-11-27

**Authors:** Lauren A Gould, Adam Vytykac, Matthew Carman, Jesse Dubey

**Affiliations:** 1 Emergency Medicine, Lakeland Regional Hospital, Lakeland, USA; 2 Emergency Medicine, Lakeland Regional Health, Lakeland, USA

**Keywords:** acute blindness, cerebral hyperperfusion syndrome, neurological symptoms due to hypomagnesemia, posterior reversible encephalopathy syndrome (pres), risk of seizure, severe hypertension, stroke mimic

## Abstract

Posterior reversible encephalopathy syndrome (PRES) is a neurologic disorder that causes acute or subacute cerebral edema, brain capillary leakage, and a hyperperfusion encephalopathy that results in presenting symptoms of headache, vision changes, seizure activity, altered mentation, nausea or vomiting, or focal neurologic deficits. We describe a case of PRES in the setting of severe hypertension presenting with total blindness and seizure activity in a patient with multiple risk factors. The patient had previous episodes of PRES in the past that led to the development of a seizure disorder. We also provide a summarization table of PRES pathophysiology, symptoms, risk factors, diagnostics, treatment, and sequelae. Early consideration and expedited diagnosis of PRES are paramount, as seizures and permanent deficits are avoidable with early treatment and intervention. Seizures occur in the majority of patients presenting with PRES and are associated with an increased risk of developing epilepsy, more so than those following strokes. Timely anti-hypertensives as well as anti-convulsant therapy form the basis of treatment for PRES, allowing for prompt treatment and hopefully avoidance of long-term sequelae, including seizure disorders and permanent neurologic deficits.

## Introduction

Posterior reversible encephalopathy syndrome (PRES) is a neurologic disorder that causes acute or subacute cerebral edema, brain capillary leakage, and hyperperfusion encephalopathy [1]. Typical presenting symptoms include headaches, vision changes, altered mental state, decreased responsiveness, or seizures, which can be focal or tonic-clonic [2]. PRES most commonly occurs in the setting of hypertension with systolic blood pressure (SBP) exceeding 160 mmHg, and there is further potential for subsequent irreversible damage when SBP is over 200 mmHg [3]. Initial brain CT imaging may be negative for acute pathology. MRI of the brain is the gold standard for diagnosing PRES, and PRES is likely underdiagnosed due to patients not receiving advanced imaging. Literature is notably lacking for reliable data to describe the prevalence of PRES in the general population; however, hypertensive encephalopathy clinically and radiographically overlaps with PRES and occurs in approximately 10%-15% of patients presenting with malignant hypertension, described as BP elevation >200/120 mmHg and associated retinopathy [4]. Risk factors for PRES include hypertension, renal disease, autoimmune diseases, immunosuppressive drugs, liver disease, and preeclampsia [1,3,5]. Seizures occur in 60%-87% of patients presenting with PRES and are associated with an increased risk of developing epilepsy, specifically a 2.9 times higher risk than those following strokes [5,6,7]. We describe a case of recurring PRES in the setting of severe hypertension presenting with total blindness and seizures in a patient with multiple risk factors. We also summarize PRES pathophysiology, symptoms, risk factors, diagnostics, treatment, and sequelae in this case report.

## Case presentation

The patient was a 49-year-old African American female with a past medical history of systemic lupus, hypertension, seizure disorder on oxcarbazepine, and end-stage renal disease secondary to lupus nephritis and hypertensive nephropathy on hemodialysis three times a week, and a history of failed kidney transplant who presented to the emergency department by emergency medical services (EMS) as a stroke alert after developing acute painless bilateral complete vision loss approximately four hours prior to arrival. During transport to the hospital, EMS applied nitropaste to the patient secondary to severe hypertension with a SBP greater than 240 mmHg. 

**Figure 1:**
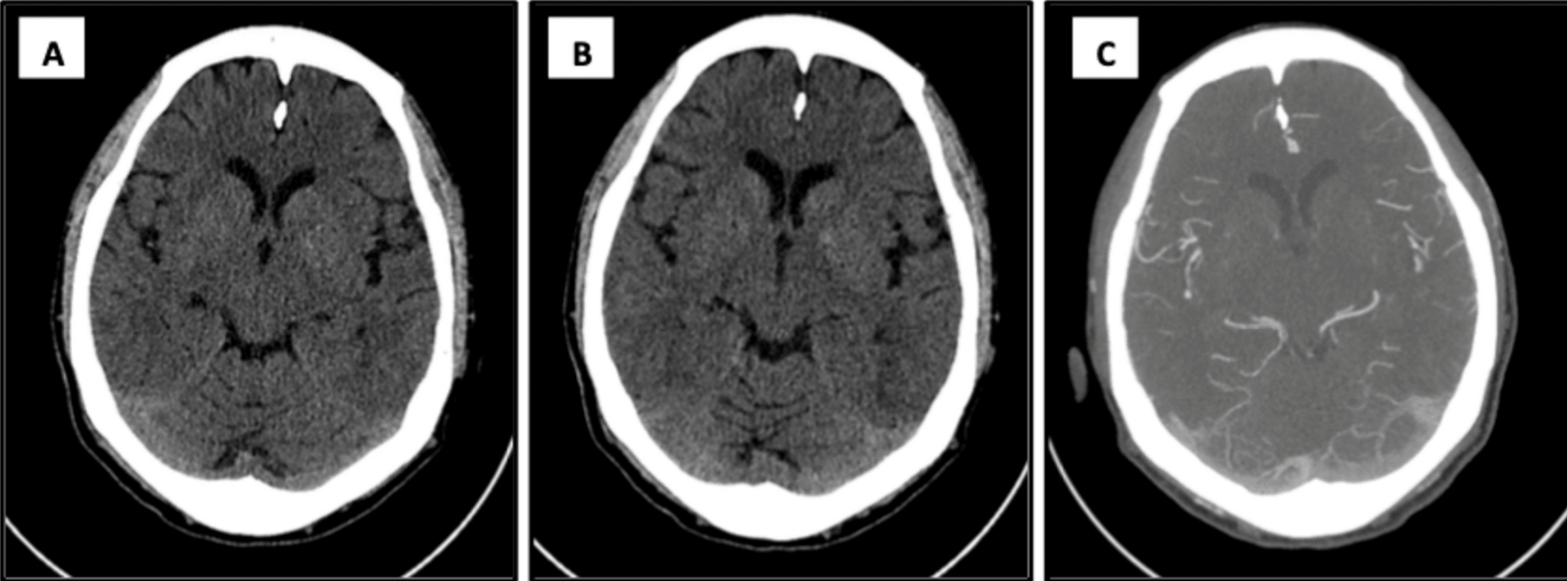
CT imaging of the brain CT without contrast (A and B) demonstrating hyperattenuation in the posterior occipital region.  No acute infarcts or masses or hemorrhages appreciated. CT angiogram of the brain (C) demonstrating similar hyperattenuation due to vasogenic edema. No stenosis or aneurysm of the cerebral vasculature is noted.

Upon arrival, the patient was experiencing total blindness with severe headache, nausea, and one episode of non-bloody emesis. Prior to undergoing CT imaging, the patient experienced a generalized tonic-clonic seizure lasting approximately one minute in duration and was subsequently treated with IV lorazepam. During the post-ictal phase, the patient was extremely agitated and required intubation for airway protection. After successful intubation, the patient was loaded with 1 g of IV Keppra, and the hyperkalemia protocol was initiated with 2 g of IV calcium gluconate, eight units of insulin, and 50 mEq of sodium bicarbonate due to a point-of-care potassium of 6.2 mmol/L. Nitropaste was discontinued in exchange for starting a nicardipine drip at 5 mg/hr, which was then titrated due to being first-line when treating a hypertensive emergency. Initial BP upon admission was markedly elevated at 242/135 mmHg. The patient was ultimately taken to a CT scan immediately upon stabilization, and the initial non-contrast CT of the head was negative for intracranial hemorrhage but did demonstrate posterior hyperattenuation concerning for PRES, as seen in Figure 1. A CT angiogram of the head and neck was negative for significant stenosis or aneurysm within the carotid and vertebral arteries and intracranial circulation. Aneurysmal dilatation of the left subclavian vein was noted. Posterior hyperattenuation was once again seen on CT angiogram (Figure 1). The patient was deemed not to be a candidate for thrombolytics by the consulting neurologist due to the patient’s seizure activity and severe hypertension. There was a high suspicion of PRES at this time, and the patient was admitted to the intensive care unit (ICU) for further evaluation and treatment.

On ICU day 1, the patient remained on a nicardipine drip with BP parameters no lower than 180/110 mmHg for the first 24 hours. The patient received initial hemodialysis and then continued on her regular schedule every Monday, Wednesday, and Friday. The patient remained on seizure precautions and received IV Keppra 1 g twice daily. An electroencephalogram (EEG) was performed that demonstrated seizure activity and lateralized periodic discharges primarily in the left hemisphere and diffuse slowing consistent with toxic/metabolic encephalopathy. An echocardiogram was performed that demonstrated a preserved ejection fraction of 55% to 60%, mild to moderate concentric left ventricular hypertrophy, normal chamber sizes, and mild tricuspid regurgitation.

**Figure 2:**
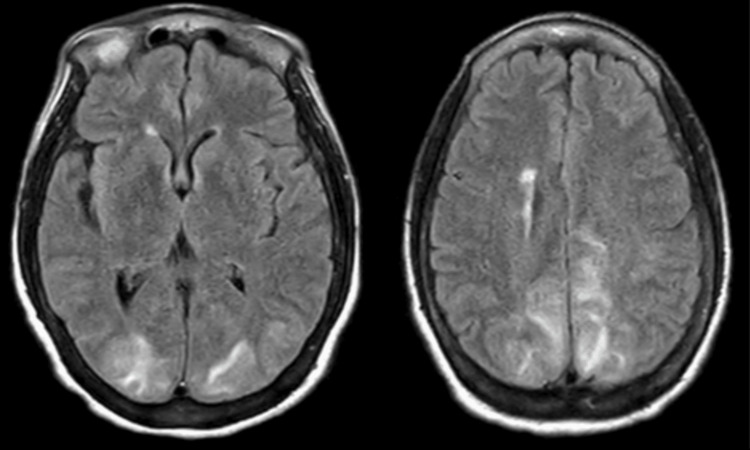
FLAIR MRI demonstrating diffuse gyral signal brightening bilaterally, most apparent posteriorly. Diffusion-weighted imaging (not shown) was negative for acute cerebral infarction. FLAIR: fluid attenuated inversion recovery

On ICU day 2, the patient was successfully extubated, and the BP was progressively decreased to below 160 mmHg. The nicardipine drip was stopped, and the patient was restarted on all home medications, including 0.1 mg of clonidine twice daily, 50 mg of hydralazine every six hours, 25 mg of carvedilol twice daily, and 60 mg of nifedipine. An MRI of the brain and brainstem was performed, which was negative for acute cerebral infarction but demonstrated diffuse gyral signal brightening bilaterally, primarily posteriorly, concerning for PRES, as seen in Figure 2.

Over the following days, the patient continued to be closely monitored with strict BP below 140 mmHg. The patient's encephalopathy and vision progressively improved, although some visual deficits remained. The patient’s symptoms continued to resolve, and she was subsequently discharged on day 5 with scheduled outpatient follow-up with the following: primary care physician, neurology, and nephrology. The patient was advised to stop her previously prescribed oxcarbazepine and was instead prescribed 1 g of Keppra twice daily for an antiepileptic and 25 mg of carvedilol twice daily in addition to her 60 mg of nifedipine once daily for antihypertensive medications. She was also given seizure precautions and strongly encouraged to follow up with the Department of Motor Vehicles (DMV) prior to driving, given her recent seizure activity, as per Florida state recommendations.

## Discussion

PRES is caused by cerebral vasogenic edema secondary to extravasation of intravascular fluid and inflammation, most commonly caused by cerebral vascular dysfunction (secondary to hypertension) or vasculature endothelial injury. This case involved a 49-year-old female with multiple risk factors who presented with acute bilateral complete vision loss and seizures secondary to acute PRES. Our patient's risk factors included hypertension, autoimmune disease secondary to lupus, lupus nephritis, end-stage renal disease, and a history of a kidney transplant with previous immunosuppressant use. Romergryko et al. reported that the incidence of PRES in lupus patients is approximately 0.7%, and Manadan et al. reported that lupus nephritis has an odds ratio (OR) of 7.53 associated with developing PRES [8,9]. The same study found female sex to have an OR of 2.28, chronic renal failure an OR of 12.1, and hypertension an OR of 8.73 [9].

While hypertension is the most commonly associated risk factor for PRES, some medications can also cause PRES, as described in Table 1. Chemotherapy agents, cytotoxic medications, or immunotherapies (i.e., tacrolimus, monoclonal antibodies) can cause toxic effects on vasculature endothelium, compromising the blood-brain barrier that may lead to extravasation of intravascular fluid and cerebral edema, causing PRES [1]. Hypomagnesemia has also been closely associated with PRES, especially in the subacute phase within 48 hours, and may contribute to neuroinflammation [1]. While our patient did not experience hypomagnesemia, her magnesium levels did downtrend within the first 24 hours of admission. Hypomagnesemia should be avoided in patients presenting with possible PRES, with experts suggesting aggressive replacement to keep levels in the high normal range ≥ 2 mg/dL [2,10]. Magnesium has known anticonvulsive and vasodilatory effects, which may be beneficial in patients presenting with PRES [2,11]. An overview of PRES pathophysiology, symptoms, risk factors, diagnostics, treatment, and possible sequelae based on current literature is briefly summarized in Table 1.

**Table 1:** An overview of posterior reversible encephalopathy syndrome (PRES) Table credits: Lauren Gould, DO (Author) CHOP: cyclophosphamide + doxorubicin + vincristine + prednisone

Pathophysiology	Symptoms	Risk Factors	Diagnostics	Treatment	Sequelae
Increased hydrostatic pressure from hyperperfusion causes extravasation of intravascular fluid, causing edema [1]; Vascular endothelial damage compromises the blood-brain barrier and leads to vascular leakage and edema [1]. Neuroinflammation may be caused by sepsis, uremia, autoimmune disorders, or hypomagnesemia [1].	Headaches, vision changes, seizures (focal or tonic-clonic), altered mental state, and decreased responsiveness [1,2].	Hypertension (most common), renal disease, autoimmune disease, immunosuppressive drugs (i.e., tacrolimus), chemotherapy (i.e, CHOP, platinum-containing medications), monoclonal antibodies, Liver disease, preeclampsia/eclampsia, and hypomagnesemia [1,3,5].	MRI of the brain (gold standard), CT of the brain, and CT angiogram of the brain; Vasogenic cerebral edema on imaging is most commonly seen posteriorly. Replace magnesium to keep levels > 2 mg/dL [2,10].	Antihypertensives (i.e., nicardipine drip), antiepileptics (i.e., Keppra), and corticosteroids (no definitive data) [1].	Epilepsy, permanent neurologic deficits (most commonly vision-related), and life-threatening cerebellar herniation [4,7,8].

The differential diagnosis of PRES includes infectious causes such as meningitis or encephalitis, intracranial hemorrhage, venous sinus thrombosis, acute ischemic stroke, demyelinating diseases, or hypertensive encephalopathy. PRES is differentiated from hypertensive encephalopathy by characteristic imaging findings such as vasogenic edema seen on MRI and potentially CT imaging, both of which were seen in our patient and described in Figures 1, 2.

After initial evaluation of the patient, it was later learned that the patient also had a history of PRES in the past and subsequently developed her seizure disorder for which she was taking oxcarbazepine. Seizures are highly associated with PRES, with 60%-87% of patients presenting with seizure activity [5,6,7]. Other sequelae of PRES include permanent neurologic deficits, including residual visual impairment as seen in our patient, and rarely cerebellar herniation.

Strict BP control is the primary treatment and preventative measure for PRES, and patients should be encouraged to monitor their BP daily, and home antihypertensive medications should be adjusted to maintain adequate control. Corticosteroids may also provide benefit by decreasing vasogenic edema, although there is no definitive data to support this currently [1]. As demonstrated by our patient, PRES can be a recurring syndrome and may result in sequelae such as seizure disorders, irreversible organ damage, and, in some cases, permanent neurologic deficits. It should be noted that this case report is limited to a single-patient experience, and information may not be generally applicable to all other patients.

## Conclusions

Early consideration and expedited diagnosis of PRES are paramount, as seizures and permanent deficits are avoidable with early treatment and intervention. PRES should especially be considered in patients with new-onset seizures and/or altered mental status, particularly in the setting of severe hypertension when initial CT imaging is negative for acute pathology and no other etiology of the patient’s symptoms (i.e., metabolic encephalopathy secondary to infection) is identified. Seizures occur in the majority of patients presenting with PRES and are associated with increased long-term seizure risk, more so than following strokes. Timely anti-hypertensives as well as anti-convulsant therapy form the basis of treatment for PRES. Additionally, addressing underlying contributing factors such as renal disease, electrolyte abnormalities, and hypomagnesemia should also be prioritized to optimize positive outcomes, allowing for prompt treatment and hopefully avoidance of long-term sequelae such as seizure disorders and permanent neurologic deficits.
